# Does the Value of FSH Predict Severity of Metabolic Complications in Females with POI?

**DOI:** 10.3390/jcm11072024

**Published:** 2022-04-05

**Authors:** Michał Kunicki, Jagoda Kruszewska, Jolanta Skórska, Hanna Laudy-Wiaderny, Marcin Wrona, Roman Smolarczyk

**Affiliations:** 1 Department of Gynecological Endocrinology, Medical University of Warsaw, 00-315 Warsaw, Poland; mkunicki@wum.edu.pl (M.K.); jolanta.skorska@gmail.com (J.S.); sicco@interia.pl (M.W.); rsmolarczyk@wum.edu.pl (R.S.); 2 INVICTA Fertility and Reproductive Center, 00-019 Warsaw, Poland; 3 Chair and Department of Experimental and Clinical Physiology, Laboratory of Centre for Preclinical Research, Medical University of Warsaw, 02-097 Warsaw, Poland; 4 Independent Public Healthcare Complex in Masovian Minsk, 05-300 Masovian Minsk, Poland; hlaudywiaderny@gmail.com

**Keywords:** POI, cardiovascular health, lipids, menopause, ovarian failure, FSH, insulin, metabolism, LDL-C, hypoestrogenism

## Abstract

Premature ovarian insufficiency (POI) is defined as a cessation of ovarian function before the age of 40. Such early deprivation of estrogens in women may be associated with several adverse cardiovascular and metabolic consequences. The aim of this retrospective study was to investigate whether women with POI and a serum follicle-stimulating hormone (FSH) level of 25–40 I/U (Group A) have the same metabolic profile as women with POI and a serum FSH level of >40 I/U (Group B). One hundred twenty-three women were included in the study group (Group A; *n* = 41; Group B; *n* = 82). The control group comprised 77 healthy women with regular menstruation. In the age- and BMI-adjusted model, no differences were found between the groups with respect to total cholesterol, high-density lipoproteins, triglycerides, HOMA-IR, glucose, and insulin. The only significant difference was found in terms of low-density lipoprotein cholesterol (LDL-C). The highest serum concentration was found in Group B, the second highest was found in Group A, and the lowest was in the controls. In conclusion, changing the threshold of FSH required to establish a POI diagnosis may have an impact on the level of serum LDL-C.

## 1. Introduction

Premature ovarian insufficiency (POI) is a disease defined, according to the guidelines of the European Society of Human Reproduction and Embryology (ESHRE), as the existence of oligo-/amenorrhea with a duration of at least four months and a serum follicle stimulation hormone (FSH) level above 25 IU/L, measured on two separate occasions at least four weeks apart [1]. The previous definition of POI, back then referred to as premature ovarian failure (POF), is still frequently used worldwide and defines the disease as the coexistence of amenorrhea but with an FSH cut-off of >40 IU/L [2,3]. The prevalence of POI varies according to the studied population and the age of the affected women, but it is estimated to reach 1% in the general population [4,5,6,7,8]. There are several possible causes that can lead to the onset of POI, including genetic, autoimmune, idiopathic, environmental, and iatrogenic etiologies [9,10,11,12,13]. The latter includes a loss of ovarian function after surgery or radio- and/or chemotherapy [14,15]. POI, accompanied by other autoimmune diseases, may constitute one of the components of autoimmune polyglandular syndrome (APS) [16]. If the causative factor is not identifiable, POI is termed spontaneous or idiopathic [17]. There are several data indicating a higher risk of ischemic heart disease and an increased mortality rate of cardiovascular origin among women diagnosed with POI. Additionally, several studies have implicated that affected females are at an increased risk of dyslipidemia and insulin resistance [18,19,20,21,22].

The aim of this study was to determine whether women with a serum FSH level of 25–40 IU/L have the same metabolic profile as women with one of >40 IU/L. The former group would not have been diagnosed as POI before 2016 and, since this time, would have fulfilled the new ESHRE criteria [1].

## 2. Materials and Methods

This is a retrospective study performed in the Department of Gynecological Endocrinology of Medical University in Warsaw. This study was approved by the Institutional Review Board of the Medical University of Warsaw (AKBE/130/2021). Informed consent was obtained from all women participating in the study regarding anonymous analysis of their medical data.

### 2.1. Inclusion and Exclusion Criteria

We searched our clinical database for data reported from January 2010 to November 2020, identifying female patients with an FSH value of >25 IU/mL during their first visit to our department (i.e., when initial diagnostic was performed). We qualified only patients with idiopathic POI. The screening was performed automatically or, when the data were not complete, manually. We excluded women who (a) had had surgery on ovaries; (b) had ever undergone chemo- or radiotherapy; (c) were administered hormonal treatment during the initial diagnostic (contraception or replacement therapy); (d) had abnormal karyotypes (e.g., Turner Syndrome); (e) were over >40 years old at the time of diagnosis establishment; f) presented an uncompensated course of systemic diseases. One hundred twenty-three POI women fulfilled the criteria and were qualified for the final analysis. Subsequently, we divided participants into two study groups based on their FSH value: POI patients with a serum FSH level of 25–40 mIU/L (Group A) and POI patients with a serum FSH level of >40 mIU/L (Group B). The median age in these groups was 36 and 30 years, respectively. The average time between the onset of menstrual disturbances and the establishment of the final diagnosis was estimated as 6–24 months. The control group comprised 77 regularly menstruating women (cycle length between 25 and 35 days). They did not suffer from any systematic diseases and did not take any medicine that could have influenced the hormonal biochemical results within six months preceding hospitalization. The median age of that group was 28 years. The control group was recruited from healthy volunteers who agreed to participate in the study or patients with regular menstruation and with no endocrine disorders stated. Afterwards, we compared the hormonal results and metabolic profiles between the three subgroups.

### 2.2. Analysis of Hormonal and Metabolic Parameters

In order to perform hormonal analysis, blood samples were taken from the patients between 8 and 10 a.m. after an overnight fast. Blood drawing occurred in the follicular phase (Days 3–5 of the menstrual cycle) in control subjects and at any convenient time in women with suspected POI. All women underwent a standard 2 h 75 g oral glucose tolerance test (OGTT), as well as a complete medical and gynecological examination. Transvaginal, transabdominal, or transrectal ultrasonography of the uterus and ovaries was performed during the luteal cycle phase in the control subjects and during the first hospitalization in POI patients.

Serum FSH, luteinizing hormone (LH), thyroid-stimulating hormone (TSH), free thyroxine (fT4), estradiol (E2), prolactin (PRL), total testosterone (T), and sex-binding hormone globulin (SHBG) were measured using an enzyme-linked fluorescent assay (ELFA) (VIDAS, bio-Mérieux, Lyon, France). 17-hydroxyprogesterone (17-OHP) levels were assessed using an enzyme-linked immunosorbent assay (ELISA) (EUROIMMUN AG Analyzer I, Wroclaw, Poland). The serum concentrations of androstenedione (ANDRO) and dehydroepiandrosterone sulfate (DHEAS) were tested using a chemiluminescent immunoassay technique (IMMULITE 2000XP, Siemens Healthineers, Erlangen, Germany). The serum insulin and cortisol (CORT) were measured using a chemiluminescent microparticle immunoassay (CMIA) (Architect i2000SR, Abbott Diagnostics, Abbott Park, IL, USA). The serum glucose, total cholesterol (TC), triglycerides (TGs), high-density lipoprotein cholesterol (HDL-C), and low-density lipoprotein cholesterol (LDL-C) were analyzed using an enzymatic colorimetric method (Konelab Prime 301 by Thermo Scientific, Waltham, MA, USA). The normal reference ranges applied in our laboratory were as follows: FSH: 3.03–8.08 mIU/mL; LH: 1.8–11.78 mIU/mL; E2: 21–251 pg/mL; PRL: 5–35 ng/mL; TSH: 0.35–4.94 μmol/mL; fT4: 9.01–19.05 pmol/L; ANDRO: 0.3–3.5 ng/mL; DHEAS: 2.68–9.23 μmol/L; T: 0.1–0.56 ng/mL; SHBG: 19.84–155.2 nmol/L; 17-OHP: 0.3–1.0 ng/mL; TC: <4.9 mmol/L; TGs: <1.71 mmol/L; HDL-C: <1.2 mmol/L; fasting glucose (GLU): 3.9–5.5 mmol/L; glucose after 2 h of OGTT (GLU 120): <7.8 mmol/L; LDL-C: <3.0 mmol/L.

Insulin resistance was calculated using the homeostatic model assessment insulin resistance index (HOMA-IR) according to the formula (HOMA-IR = [fasting insulin fasting glucose]/22.5). Insulin resistance was ascertained when HOMA-IR > 2.5 [21].

The body mass index (BMI) was calculated as weight divided by height squared and expressed in kg/m^2^. The free androgen index (FAI) was calculated as 100 × (T/SHBG).

Ultrasonography of the uterus and ovaries was performed with the transducer Aloka Alpha7.

### 2.3. Statistical Analysis

The Shapiro–Wilk test was performed to verify the normality of the data distribution, and Levene’s test for the homogeneity of variances was applied. The differences between the three groups were checked using non-parametric ANOVA (Kruskal–Wallis). In the case of significant results (*p* < 0.05), post hoc tests (Dunn test) were performed for comparisons between pairs of groups. The chi-squared test with Bonferroni corrections was performed to compare the categorical variables. The comparison of median values of metabolic parameters between the three groups was adjusted for BMI and age. If the initial assumptions were fulfilled, a linear model of regression was built; if not, either the dependent variable was log-transformed or, subsequently, another reasoning was undertaken. Correlation analysis was performed using Spearman’s rank-order correlation.

StatSoft 2012 STATISTICA Version 13 (Cracow, Poland) was used for statistical calculations. A *p*-value of <0.05 was considered statistically significant.

## 3. Results

### 3.1. Comparison of Hormonal Profile, Age, and BMI among Study and Control Groups

The detailed characteristics of the study and control groups, together with their hormonal results, are presented in Table 1. The data indicated that there were significant differences between age and BMI among the three groups. The oldest women were found in Group A, then in Group B, and then the youngest women in the controls. (*p* < 0.05). The women in Group A also had the highest BMI levels.

Patients with POI (both Group A and Group B) had higher FSH and LH and lower estradiol in comparison to the controls, which is in line with the definition of POI. Patients with ovarian insufficiency also had significantly lower serum androgens and cortisol levels. Because both age and BMI could influence the biochemical (metabolic) parameters, further analysis was conducted after adjusting variables for these parameters.

### 3.2. Comparison of Metabolic Profile among Study and Control Groups

In the age- and BMI-adjusted model, no differences were found between the groups with respect to serum TC, HDL-C, and TGs concentration. The only significant difference was found in LDL-C. The highest serum concentration was found among patients within Group B, followed by Group A; the lowest level was detected in the control subjects. Regardless of confounding factors, when compared to the control subjects, the LDL-C value increased by 41.2% and 24.8% in Group B and Group A, respectively.

There were no differences between groups with respect to HOMA-IR, glucose, and insulin, both in a fasting state and after a 75 g glucose load.

Comprehensive data for metabolic profile comparisons between groups are presented in Table 2.

### 3.3. Comparison of Proportions of Abnormal Results between the Groups

The highest percentage of women with a HOMA of >2.5 was found in Group B (11.7%), followed by Group A (4.9%); the lowest was found in the controls (2.7%). However, the difference did not reach statistical significance between the three groups (*p* = 0.078). There was also no difference in the number of women with elevated total cholesterol (*p* = 0.073). The highest percentage of women with elevated cholesterol was found in Group A (51.2%), followed by Group B (39%) and the controls (29.2%).

Contrarily, there was a significantly higher percentage of females with increased above the upper limit of normal range serum TGs and LDL-C in Groups A and B in comparison to the control subjects (but not between Group A and Group B). An elevated serum LDL-C level was found in the following proportions within the subsequent groups: 34.1% (Group B), 31.7% (Group A), and 5.2% (Control). In terms of TGs, these parameters were calculated as 12.2% (Group B), 9.8% (Group A), and 1.3% (Control). Statistically significant data are presented in Table 3 and Table 4.

### 3.4. Analysis of Correlations

INS 0, HOMA-IR, and LDL-C correlated positively with FAI in Group A. Similarly, HOMA, INS 0, INS 60, and INS 120 correlated with FAI in Group B. The positive correlation with respect to LDL-C in Group B did not reach significance.

The correlations between the measured parameters are shown in Table 5 and Table 6.

## 4. Discussion

The main goal of this study was to compare the metabolic profile of POI women with an FSH level of 25–40 IU/L with the subgroup of affected individuals with an FSH level of >40 IU/L. As we stated before, the previous definition, which is still frequently encountered and applied, established an FSH threshold of >40 IU/L required for POI diagnosis. However, in 2016, the European Society of Human Reproduction and Embryology (ESHRE) recommended decreasing the cut-off value of FSH. According to the consensus, POI can be diagnosed when FSH reaches >25 I/U, based on two different measurements >4 weeks apart [1]. Both of these definitions are the most common in medical studies, but the National Institute for Health and Care Excellence (NICE) has suggested adopting an FSH level of >30 IU/L as the threshold for POI detection [23]. Therefore, we wondered whether women who would not have been diagnosed with POI before 2015 (i.e., having a serum FSH level of 25–40 IU/L) differ with respect to metabolic profiles from patients with an FSH level of >40 IU/L.

To the best of our knowledge, this is the first study that compares metabolic profiles in women with POI considering these different thresholds. POI development may be credited to several pathogenic (e.g., genetic, iatrogenic, and metabolic) factors, but we only focused on spontaneous POI in karyotypically normal women. Firstly, the available data indicate that idiopathic POI may account for 65% of cases [24]. Additionally, there is evidence that an abnormal karyotype can also influence serum lipid levels [25].

POI is associated with metabolic abnormalities and an increased risk of cardiovascular diseases [26,27,28,29,30]. There is agreement that it occurs due to the hypoestrogenism found in individuals with POI [31]. Increased mortality of cardiovascular origin has also been reported in patients with POI in comparison to healthy postmenopausal women [32,33,34,35,36]. It is also widely known that increased incidence of cardiovascular events and mortality occurs with an abnormal lipid profile [37]. Besides lipids, insulin resistance and abnormal glucose levels could also play a role [38]. Generally, POI is associated with insulin resistance and a higher risk of the development of type 2 diabetes mellitus, although contrary results also exist [7,39]. Hyperinsulinemia, by decreasing lipoprotein lipase activity, leads to increase serum TGs levels, which is an independent cardiovascular risk factor [7]. In conclusion, it may be stated that an abnormal lipid profile and insulin resistance may both promote the incidence of cardiovascular events.

In our study, no statistical differences were stated between the groups with respect to total cholesterol, high-density lipoprotein cholesterol, and triglycerides. As we presented, the only significant difference was found in terms of LDL-C. The highest serum concentration was detected in POI individuals with an FSH level of >40 IU/L, followed by POI patients with an FSH level of 25–40 IU/L, and the lowest was found in the healthy controls. We did not observe any differences with respect to HOMA-IR, fasting blood glucose, or OGTT between the groups. Our results suggest that decreasing the cut-off level of FSH to 25 IU/L (in line with the latest POI definition) has no impact on glucose or lipid metabolism, but it does impact serum LDL-C concentration. This parameter was highest in women with an FSH level of >40 IU/L. Because the time from the beginning of amenorrhea to admission to the clinic was similar between studies, and no significant differences were found in terms of hormonal results (e.g., estradiol and TSH concentration) after adjusting data to age and BMI, other factors could probably play a role.

Harchaoui et al. found that even a slight increase in the basal FSH level in women (defined as FSH > 7 IU/L) may be associated with a greater serum cholesterol concentration [7]. Similar results were obtained by Song et al., who performed the study on postmenopausal women. They found that women with a serum FSH level of ≥78.3 IU/L had higher serum TC and LDL-C levels in comparison to those with FSH levels between 40 and 78.3 IU/L [40]. Thus, in our study, elevated serum LDL-C could be the result of increased FSH in the study groups. The pathomechanism was explained by Song et al., who proposed that signaling via the intrahepatic FSH receptor may downregulate the number of LDL-C receptors (LDLR), contributing to impaired LDL-C uptake via hepatocytes, consequently leading to an increased serum LDL-C level [40]. We agree that the same mechanism may constitute a link between increased FSH and LDL-C concentration in our patients with POI.

Several conducted studies have assessed the lipid and metabolic profile in POI patients, and their results are contradictory [41,42,43,44,45].

The metabolic profile of women diagnosed according to the new POI definition was only presented in the study by Podfigurna et al. [41]. They found a significantly higher serum TC and HDL-C and LDL-C in the POI group in comparison to the controls. Similar to our results, they did not find any significant differences between groups with respect to serum TGs, glucose, insulin concentrations, or HOMA-IR.

Kalantaridou et al. found no significant difference with respect to fasting serum glucose, TC, HDL-C, LDL-C, or TGs levels [42].

Knauff et al. compared serum lipid profiles between 90 women with POI and 198 age- and BMI-matched control subjects and found significantly higher levels of TGs and a statistically lower level of HDL-C (although values were within the reference range) in the affected individuals. The authors did not provide any information regarding the karyotypes of the included women. Additionally, a significant correlation was found between the HDL-C concentration and serum testosterone, SHBG, FAI, and TGs [43].

Gulhan et al. found significantly higher TC and LDL-C concentrations in 47 POI women compared to 60 healthy female controls. However, no difference was found between the groups with regard to TGs and HDL-C levels. They found a significant negative correlation between E2 and TC levels in the POI group, while no correlation was found between E2 and the lipid profile in the control group [45].

Ates et al. also analyzed that TC and HDL-C were higher in women with POI. There were no differences in glucose, insulin, HOMA-IR, low-density lipoprotein cholesterol, and triglyceride levels between the two groups [44].

A recent meta-analysis performed by Wang et al., which compared lipid profiles between 458 patients with POI and 551 controls, showed that serum TC, LDL-C, and TGs levels were significantly higher in POI patients when compared with healthy controls. However, serum HDL-C levels did not vary significantly between controls and POI patients [35].

We may conclude that the studies assessing the lipid and metabolic profiles of patients with POI are contradictory. While some of them found significant abnormalities in comparison to healthy subjects, others did not report such changes. In most of them, a small number of cases were included, or POI without a spontaneous origin was not excluded. In some studies, the authors did not present data regarding karyotype status. Therefore, we cannot compare our data to other similar studies.

Our study has limitations as well. We did not record all of the confounders that could have influenced the results, e.g., smoking habits. Secondly, we adjusted data for BMI and age, but probably there are other factors that should have been considered, such as an increased rate of hypoandrogenism in the study groups. Furthermore, our study is retrospective, which can be a source of bias. We also did not report detailed data on the time interval between the last menstruation and the diagnosis; nevertheless, in each case, it was no longer than 6–24 months.

However, this is the first study that compares metabolic profiles in two defined groups of patients. Another advantage of this study is the fact that the group was homogeneous and large. Moreover, we excluded patients with POI due to iatrogenic or genetic etiologies, as such individuals have additional cardiovascular risk factors.

There is a need for further research on metabolic complications among POI individuals. Most of the above-mentioned studies had a retrospective design and comprised a small number of participants; therefore, future studies of a prospective nature would be particularly valuable, as well as those with a greater sample size.

## 5. Conclusions

Our study is the first to compare metabolic profiles in two separate subgroups of individuals with premature ovarian insufficiency diagnosed based on different cut-off levels of FSH. We may conclude that changing the threshold of FSH required for establishing POI diagnosis has an impact on serum LDL-C cholesterol level. We observed that POI patients with an FSH level of >40 IU/L had a 41.2% higher LDL-C concentration in comparison to the controls, and the LDL-C concentration of patients with an FSH level of 25–40 IU/L was 24.8% higher. Most studies in this field remain contradictory, so further research is required to understand the prevalence of metabolic complications among individuals with premature ovarian insufficiency.

## Figures and Tables

**Table 1:** Hormonal and clinical features of patients with POI and the control group.

Parameter	Group A (FSH 25–40; *n* = 41)	Group B (FSH > 40; *n* = 82)	Control (*n* = 77)	*p*-Value
age (year)	36 (30–39)	30 (27–34)	28 (24–32)	0.0000
BMI (kg/m^2^)	24.00 (22.5–28.5)	22.50 (21–24.9)	22.75 (20.5–25)	0.0077
FSH (mIU/mL)	30.76 (28.5–34.01)	67.70 (51–85.36)	5.30 (4.2–5.97)	0.0000
LH (mIU/mL)	16.88 (13.83–25.75)	32.00 (23.26–39.36)	4.66 (3.5–6.11)	0.0000
E2 (pg/mL)	24.50 (14.5–47.5)	13.55 (10–25)	40.00 (29–52)	0.0000
PRL (ng/mL)	22.90 (13.365–29.98)	24.83 (16.07–32.42)	25.22 (18.01–34.1)	0.5137
T (ng/mL)	0.30 (0.23–0.35)	0.32 (0.23–0.42)	0.36 (0.29–0.43)	0.0095
SHBG (nmol/L)	58.00 (39.5–95.9)	57.05 (39.35–73.85)	61.10 (45.3–88.9)	0.3031
FAI	0.51 (0.316–0.656)	0.56 (0.340–0.856)	0.55 (0.394–0.836)	0.2896
ANDRO (ng/mL)	1.64 (1.31–2.28)	2.00 (1.5–2.8)	2.78 (2.1–3.1)	0.0000
DHEAS (μmol/L)	5.25 (3.61–7.54)	5.80 (4.14–6.43)	6.79 (5.23–8.43)	0.0005
17-OHP (ng/mL)	1.28 (0.79–1.89)	0.78 (0.548–1.08)	1.27 (0.82–1.87)	0.0000
TSH (mIU/L)	1.33 (0.995–2.136)	1.34 (1–1.59)	1.44 (0.9–2.1)	0.5786
fT4 (pmol/L)	12.74 (11.625–13.665)	13.21 (11.62–14.17)	13.21 (12.04–13.86)	0.3605
CORT 8 a.m. (μg/dL)	12.07 (10.3–15.6775)	11.06 (9.349–14.77)	13.88 (12.129–15.621)	0.0061

Data of variables are expressed as median value (25–75 quartiles), statistically significant results are marked in color; POI—premature ovarian insufficiency; BMI—body mass index; FSH—follicle stimulating hormone; LH—luteinizing hormone; E2—estradiol; PRL—prolactin; T—total testosterone; SHGB—sex hormone binding globulin; FAI—free androgen index; ANDRO—androstenedione; DHEA-S—dehydroepiandrosterone sulfate; 17-OH-P—17-hydroxyprogesterone; TSH—thyroid stimulating hormone; fT4—free thyroxine, CORT—cortisol.

**Table 2:** Metabolic profile of patients with POI and the control group.

Parameter	Group A (FSH 25–40; *n* = 41)	Group B (FSH > 40; *n* = 82)	Control (*n* = 77)	*p*-Value (after Adjustment to Age and BMI)
TC (mmol/L)	4.92 (4.2–5.52)	4.66 (4.17–5.46)	4.63 (4.01–4.94)	*p* = 0.081
HDL (mmol/L)	1.53 (1.4–1.91)	1.55 (1.3–1.86)	1.78 (1.5–2.46)	*p* = 0.898
LDL (mmol/L)	2.49 (1.99–3.35)	2.60 (2.15–3.21)	1.84 (1.45–2.22)	*p* = 0.016
TG (mmol/L)	0.91 (0.72–1.21)	0.80 (0.59–1.28)	0.80 (0.63–0.97)	*p* = 0.149
HOMA	1.24 (0.98–1.57)	1.12 (0.77–1.8)	1.11 (0.86–1.39)	*p* = 0.864
GLU (mmol/L)	4.94 (4.77–5.16)	4.79 (4.55–5.06)	4.67 (4.44–4.83)	*p* = 0.211
GLU 120 (mmol/L)	5.50 (4.55–6.39)	5.21 (4.44–6.5)	5.36 (4.28–6.17)	*p* = 0.975
INS 0 (IU/mL)	5.70 (4.5–7.1)	5.30 (3.9–8.4)	5.50 (4.1–6.8)	*p* = 0.873
INS 60 (IU/mL)	37.55 (25.10–63.80)	40.30 (26.8–71.8)	42.10 (25.7–57.7)	*p* = 0.376
INS 120 (IU/mL)	26.30 (16.5–36.9)	29.10 (17.7–43.9)	30.60 (20.9–52.4)	*p* = 0.145

Data of variables are expressed as median value (25–75 quartiles), statistically significant results are marked in color; POI—premature ovarian insufficiency; TC—total cholesterol; HDL—high-density lipoprotein cholesterol; LDL—low-density lipoprotein cholesterol; TG—triglycerides; HOMA-IR—Homeostatic Model Assessment-Insulin Resistance; GLU—fasting glucose; GLU 120—glucose measured 120 min after a 75 g oral glucose loading; INS—fasting insulin; INS60—insulin measured 60 min after a 75 g oral glucose loading; INS120—insulin measured 120 min after a 75 g oral glucose loading.

**Table 3:** The number and percentage of women with elevated LDL-C levels.

	Group A (FSH 25–40; *n* = 41)	Group B (FSH > 40; *n* = 82)	Control (*n* = 77)
Number of women with normal LDL level	28	54	73
Number of women with elevated LDL level	13	28	4
Percentage of abnormal results (%)	31.7	34.1	5.2

*p*-value of chi-squared test: *p* = 0.000020; post hoc analysis with Bonferroni correction between pairs of groups: control vs. Group A (*p* < 0,001); Control vs. Group B (*p* < 0.001); Group A vs. Group B (*p* = 0.787).

**Table 4:** The number and percentage of women with elevated TGs levels.

	Group A (FSH 25–40; *n* = 41)	Group B (FSH > 40; *n* = 82)	Control (*n* = 77)
Number of women with normal TG level	37	72	76
Number of women with elevated TG level	4	10	1
Percentage of abnormal results (%)	9.8	12.2	1.3

*p*-value of chi-squared test: *p* = 0,028; post hoc analysis with Bonferroni correction between pairs of groups: control vs. Group A (*p* < 0.030); Control vs. Group B (*p* < 0.007); Group A vs. Group B (*p* = 0.688).

**Table 5:** Spearman’s correlation between BMI, biochemical parameters, and hormonal results in Group A.

	FSH	LH	E2	FAI	T	DHEAS	ANDRO
**BMI**	−0.188	0.029	−0.043	0.461	−0.029	0.146	−0.045
**HOMA-IR**	0.082	−0.016	−0.007	0.514	−0.011	0.242	0.107
**GLU**	0.312	−0.076	−0.117	−0.033	−0.176	−0.042	−0.093
**GLU 120**	0.010	−0.006	−0.076	0.016	−0.252	0.105	−0.022
**INS 0**	0.010	−0.027	0.010	0.554	0.000	0.234	0.140
**INS 60**	−0.054	0.152	0.012	0.302	0.089	0.127	0.171
**INS 120**	−0.284	0.024	0.082	0.118	−0.101	0.161	0.154
**TC**	−0.140	−0.003	−0.157	0.191	0.050	0.087	0.073
**LDL**	−0.059	−0.083	−0.183	0.378	−0.053	0.121	0.069
**HDL**	0.128	0.087	0.158	−0.411	0.207	−0.105	0.076
**TG**	−0.250	0.094	0.074	0.190	0.009	0.124	0.022

Data are expressed as correlation coefficient *r*-values. Color values express statistical significance (*p* < 0.05).

**Table 6:** Spearman’s correlation between BMI, biochemical parameters, and hormonal results in Group B.

	FSH	LH	E2	FAI	T	DHEAS	ANDRO
**BMI**	−0.231	0.010	−0.147	0.089	0.008	0.107	−0.106
**HOMA-IR**	−0.026	−0.240	−0.077	0.384	0.122	0.135	−0.039
**GLU**	0.154	0.010	0.020	0.130	0.003	−0.008	−0.066
**GLU 120**	0.001	−0.115	−0.076	0.158	−0.023	−0.082	−0.192
**INS 0**	−0.040	−0.237	−0.105	0.381	0.118	0.140	−0.046
**INS 60**	−0.121	−0.162	−0.171	0.328	0.124	0.160	−0.007
**INS 120**	−0.023	−0.128	−0.134	0.326	0.102	0.138	−0.012
**TC**	−0.076	−0.083	−0.073	0.100	0.174	0.110	0.037
**LDL**	−0.235	−0.233	−0.056	0.204	0.118	0.095	0.030
**HDL**	0.091	0.119	0.098	−0.293	−0.051	−0.023	0.017
**TG**	−0.248	−0.242	0.014	0.083	0.107	−0.265	−0.015

Data are expressed as correlation coefficient *r*-values. Color values express statistical significance (*p* < 0.05).

## Data Availability

Not applicable.

## References

[1] Webber L., Davies M., Anderson R., Bartlett J., Braat D., Cartwright B., Cífková R., Keizer-Schrama S.D.M., Hogervorst E., Janse F. (2016). ESHRE Guideline: Management of women with premature ovarian insufficiency. Hum. Reprod..

[2] Nelson L.M. (2009). Primary Ovarian Insufficiency. N. Engl. J. Med..

[3] Haller-Kikkatalo K., Uibo R., Kurg A., Salumets A. (2015). The prevalence and phenotypic characteristics of spontaneous premature ovarian failure: A general population registry-based study. Hum. Reprod..

[4] Coulam C.B., Adamson S.C., Annegers J.F. (1986). Incidence of Premature Ovarian Failure. Obstet. Gynecol. Surv..

[5] Chang S.H., Kim C.-S., Lee K.-S., Kim H., Yim S.V., Lim Y.J., Park S.K. (2007). Premenopausal factors influencing premature ovarian failure and early menopause. Maturitas.

[6] Lagergren K., Hammar M., Nedstrand E., Bladh M., Sydsjö G. (2018). The prevalence of primary ovarian insufficiency in Sweden; a national register study. BMC Women's Health.

[7] Harchaoui K.E.L., Visser M.E., Kastelein J.J.P., Stroes E.S., Dallinga-Thie G.M. (2009). Triglycerides and Cardiovascular Risk. Curr. Cardiol. Rev..

[8] Bakalov V.K., Anasti J.N., Calis K.A., Vanderhoof V.H., Premkumar A., Chen S., Furmaniak J., Smith B.R., Merino M.J., Nelson L.M. (2005). Autoimmune oophoritis as a mechanism of follicular dysfunction in women with 46, XX spontaneous premature ovarian failure. Fertil. Steril..

[9] Panay N., Anderson R.A., Nappi R.E., Vincent A.J., Vujovic S., Webber L., Wolfman W. (2020). Premature ovarian insufficiency: An International Menopause Society White Paper. Climacteric.

[10] Vujovic S., Brincat M., Erel C.T., Gambacciani M., Lambrinoudaki I., Moen M.H., Schenck-Gustafsson K., Tremollieres F., Rozenberg S., Rees M. (2010). EMAS position statement: Managing women with premature ovarian failure. Maturitas.

[11] Fenton A.J. (2015). Premature ovarian insufficiency: Pathogenesis and management. J. Mid-Life Health.

[12] Tucker E., Grover S.R., Bachelot A., Touraine P., Sinclair A. (2016). Premature Ovarian Insufficiency: New Perspectives on Genetic Cause and Phenotypic Spectrum. Endocr. Rev..

[13] Chapman C., Cree L., Shelling A.N. (2015). The genetics of premature ovarian failure: Current perspectives. Int. J. Women's Health.

[14] Silva C., Yamakami L., Aikawa N., Araujo D., Carvalho J., Bonfá E. (2014). Autoimmune primary ovarian insufficiency. Autoimmun. Rev..

[15] Ebrahimi M., Asbagh F.A. (2011). Pathogenesis and Causes of Premature Ovarian Failure: An Update. Int. J. Fertil. Steril..

[16] Szlendak-Sauer K., Jakubik D., Kunicki M., Skórska J., Smolarczyk R. (2016). Autoimmune polyglandular syndrome type 3 (APS-3) among patients with premature ovarian insufficiency (POI). Eur. J. Obstet. Gynecol. Reprod. Biol..

[17] Rossetti R., Ferrari I., Bonomi M., Persani L. (2017). Genetics of primary ovarian insufficiency. Clin. Genet..

[18] Jensen J., Nilas L., Christiansen C. (1990). Influence of menopause on serum lipids and lipoproteins. Maturitas.

[19] Abdulnour J., Doucet É., Brochu M., Lavoie J.-M., Strychar I., Rabasa-Lhoret R., Prud’Homme D. (2012). The effect of the menopausal transition on body composition and cardiometabolic risk factors: A montreal-ottawa new emerging team group study. Menopause.

[20] Daan N.M.P., Muka T., Koster M.P.H., Van Lennep J.E.R., Lambalk C.B., Laven J.S.E., Fauser C.G.K.M., Meun C., De Rijke Y.B., Boersma E. (2016). Cardiovascular Risk in Women with Premature Ovarian Insufficiency Compared to Premenopausal Women at Middle Age. J. Clin. Endocrinol. Metab..

[21] Kunicki M., Rudnicka E., Skórska J., Calik-Ksepka A.I., Smolarczyk R. (2018). Insulin resistance indexes in women with premature ovarian insufficiency–A pilot study. Ginekol. Polska.

[22] Kim C.J., Kim T.H., Ryu W.S., Ryoo U.H. (2000). Influence of menopause on high density lipoprotein-cholesterol and lipids. J. Korean Med. Sci..

[23] NICE|Menopause: Diagnosis and Management|Guidance and Guidelines|NICE. https://www.nice.org.uk/guidance/ng23.

[24] Moreira A.M., Spritzer P.M. (2016). Primary ovarian insufficiency: Different approaches in three cases and a review of literature. Endocrinol. Diabetes Metab. Case Rep..

[25] Lebenthal Y., Levy S., Sofrin-Drucker E., Nagelberg N., Weintrob N., Shalitin S., De Vries L., Tenenbaum A., Phillip M., Lazar L. (2018). The Natural History of Metabolic Comorbidities in Turner Syndrome from Childhood to Early Adulthood: Comparison between 45, X Monosomy and Other Karyotypes. Front. Endocrinol..

[26] van Lennep J.E.R., Heida K.Y., Bots M.L., Hoek A. (2016). Cardiovascular disease risk in women with premature ovarian insufficiency: A systematic review and meta-analysis. Eur. J. Prev. Cardiol..

[27] Mishra G.D., Chung H.-F., Cano A., Chedraui P., Goulis D.G., Lopes P., Mueck A., Rees M., Senturk L.M., Simoncini T. (2019). EMAS position statement: Predictors of premature and early natural menopause. Maturitas.

[28] Panay N., Stevenson J.C. (2019). Premature Ovarian Insufficiency and Long-Term Health Consequences. Curr. Vasc. Pharmacol..

[29] Collins P., Webb C., De Villiers T.J., Stevenson J.C., Panay N., Baber R. (2016). Cardiovascular risk assessment in women–An update. Climacteric.

[30] Stevenson J.C., Collins P., Hamoda H., Lambrinoudaki I., Maas A.H.E.M., Maclaran K., Panay N. (2021). Cardiometabolic health in premature ovarian insufficiency. Climacteric.

[31] Bidet M., Bachelot A., Bissauge E., Golmard J.L., Gricourt S., Dulon J., Coussieu C., Badachi Y., Touraine P. (2011). Resumption of Ovarian Function and Pregnancies in 358 Patients with Premature Ovarian Failure. J. Clin. Endocrinol. Metab..

[32] Wu X., Cai H., Kallianpur A., Li H., Yang G., Gao J., Xiang Y.-B., Ji B.-T., Tang Y., Zheng W. (2014). Impact of Premature Ovarian Failure on Mortality and Morbidity among Chinese Women. PLoS ONE.

[33] Muka T., Oliver-Williams C., Kunutsor S., Laven J.S.E., Fauser B.C.J.M., Chowdhury R., Kavousi M., Franco O. (2016). Association of Age at Onset of Menopause and Time Since Onset of Menopause with Cardiovascular Outcomes, Intermediate Vascular Traits, and All-Cause Mortality: A Systematic Review and Meta-Analysis. JAMA Cardiol..

[34] Tao X.-Y., Zuo A.-Z., Wang J.-Q., Tao F.-B. (2016). Effect of primary ovarian insufficiency and early natural menopause on mortality: A meta-analysis. Climacteric.

[35] Wang Z., Fang L., Wu Z., Li Y., Jia Q., Cheng J.-C., Sun Y.-P. (2021). A meta-analysis of serum lipid profiles in premature ovarian insufficiency. Reprod. Biomed. Online.

[36] Jacobsen B.K., Knutsen S.F., Fraser G.E. (1999). Age at Natural Menopause and Total Mortality and Mortality from Ischemic Heart Disease: The Adventist Health Study. J. Clin. Epidemiology.

[37] Lemieux I., Lamarche B., Couillard C., Pascot A., Cantin B., Bergeron J., Dagenais G., Després J. (2011). Total Cholesterol/HDL Cholesterol Ratio vs LDL Cholesterol/HDL Cholesterol Ratio as Indices of Ischemic Heart Disease Risk in Men: The Quebec Cardiovascular Study. Arch. Intern. Med..

[38] Adeva-Andany M.M., Martínez-Rodríguez J., González-Lucán M., Fernández-Fernández C., Castro-Quintela E. (2019). Insulin Resistance Is a Cardiovascular Risk Factor in Humans. Diabetes Metab. Syndr. Clin. Res. Rev..

[39] Anagnostis P., Christou K., Artzouchaltzi A.-M., Gkekas N., Kosmidou N., Siolos P., A (2019). Paschou, S.; Potoupnis, M.; Kenanidis, E.; Tsiridis, E.; et al. Early menopause and premature ovarian insufficiency are associated with increased risk of type 2 diabetes: A systematic review and meta-analysis. Eur. J. Endocrinol..

[40] Song Y., Wang E.-S., Xing L.-L., Shi S., Qu F., Zhang D., Li J.-Y., Shu J., Meng Y., Sheng J.-Z. (2016). Follicle-stimulating hormone induces postmenopausal dyslipidemia through inhibiting hepatic cholesterol metabolism. J. Clin. Endocrinol. Metab..

[41] Podfigurna A., Stellmach A., Szeliga A., Czyzyk A., Meczekalski B. (2018). Metabolic Profile of Patients with Premature Ovarian Insufficiency. J. Clin. Med..

[42] Kalantaridou S.N., Naka K., Papanikolaou E., Kazakos N., Kravariti M., Calis K.A., Paraskevaidis E.A., Sideris D.A., Tsatsoulis A., Chrousos G.P. (2004). Impaired Endothelial Function in Young Women with Premature Ovarian Failure: Normalization with Hormone Therapy. J. Clin. Endocrinol. Metab..

[43] Knauff E.A., Westerveld H.E., Goverde A.J., Eijkemans M.J., Valkenburg O., van Santbrink E.J., Fauser B.C., van der Schouw Y.T. (2008). Lipid profile of women with premature ovarian failure. Menopause.

[44] Ates S., Yesil G., Sevket O., Molla T., Yildiz S. (2014). Comparison of metabolic profile and abdominal fat distribution between karyotypically normal women with premature ovarian insufficiency and age matched controls. Maturitas.

[45] Gulhan I., Bozkaya G., Uyar I., Oztekin D., Pamuk B.O., Dogan E. (2012). Serum lipid levels in women with premature ovarian failure. Menopause.

